# NEXAFS Spectra Simulations of Nitrogen-Bearing Heterocycles

**DOI:** 10.1021/acsomega.4c07024

**Published:** 2024-10-16

**Authors:** Ricardo R. Oliveira, Amanda D. Torres, Alexandre B. Rocha

**Affiliations:** Chemistry Institute, Federal University of Rio de Janeiro, Rio de Janeiro, Brazil - 21941-909

## Abstract

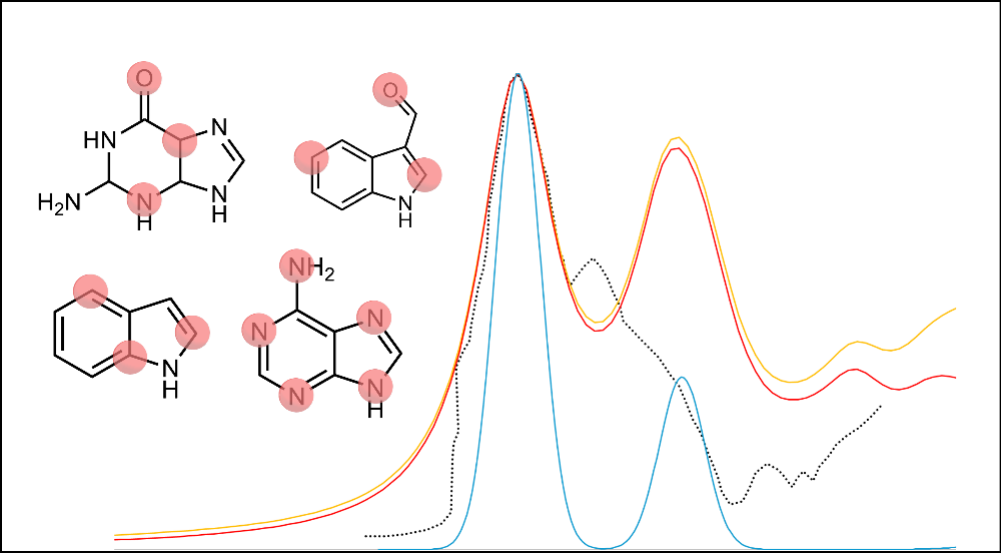

Five-membered heterocyclic compounds containing nitrogen atoms are important biomolecule building blocks. In addition to their fundamental biological importance, these molecular structures are used in several technological applications. Consequently, it is essential to develop techniques that allow the characterization of these fundamental systems. We address this issue by performing simulations of K-edge NEXAFS spectra by applying a time-dependent density functional theory (TDDFT) and an inner-shell multiconfigurational self-consistent field (IS-MCSCF) of selected molecules. Also, vibronic coupling simulations were considered for the TDDFT computations. Surprisingly, molecular orbital binding energies do not reproduce the order of the transition energies obtained by IS-MCSCF, indicating a possible breakdown of the orbital picture concerning the NEXAFS spectrum. In general, the TDDFT and IS-MCSCF results are compatible and are in close agreement with experimental data. Moreover, vibronic coupling and vertical transition results were very similar. Finally, it is important to mention that, to the best of our knowledge, this is the first time that the IS-MCSCF method has been applied to molecular systems of this size.

## Introduction

Nitrogen-bearing heterocycles are present in several important biological molecules and drugs. Illustrative examples are adenine and guanine, which are present in DNA. Basically, adenine and guanine are purine derivatives composed of a fused pyrimidine-imidazole backbone.^1^ Moreover, the pyrimidine derivative molecules cytosine and thymine are also present in the DNA structure, highlighting the fundamental role of aromatic nitrogen heterocyclic molecules in biochemistry and life sciences.^1^ Another remarkable nitrogen heterocycle is indole, which is present on several organisms, such as bacteria.^2^ It is also present in the essential amino acid tryptophan. In turn, this amino acid has important metabolic functions, and it is imperative for protein synthesis.^3^

In addition to the fundamental relevance of nitrogen heterocycles, there are several important applications. Imidazole, a five-membered heterocyclic compound, and its derivatives are good corrosion inhibitors for iron and copper due to their capacity to form stable and strongly bonded species on their surface.^4,5^ Consequently, several works analyzing the molecule–surface bonding were published.^4−6^ In coordination and organometallic chemistry, this class of molecules is very versatile. Two important examples are pyridine and bipyridine, mono- and chelating ligands, respectively.^7^ Several transition metal complexes can be formed by a combination of nitrogen heterocycle ligands exhibiting different reactivities and catalytic activities.^7^

Due to the great importance and relevance of nitrogen heterocycles, several spectroscopic works on this class of molecules were published. In particular, the near-edge X-ray absorption fine structure (NEXAFS) and X-ray photoelectron spectroscopies (XPS) are useful techniques for chemical characterization and radiation damage studies in polycyclic aromatic molecules.^8,9^ Some examples of nitrogen-bearing heterocycles that were studied by core-level techniques are indole,^10−13^ adenine,^10^ guanine,^11^ pyrimidine,^14^ purine,^14^ and their respective nucleotides.^15,16^

For complex molecules, the interpretation of core excited electronic spectra is not straightforward. Consequently, several methodologies emerged, in particular, those based on single reference methods, such as the time-dependent density functional theory (TDDFT) and coupled cluster (CC) and an algebraic-diagrammatic construction (ADC) scheme.^17,18^ A low-cost method for XPS and NEXAFS spectra simulations is TDDFT,^19−21^ giving satisfactory results for large systems. For instance, in this level of theory it was possible to qualitatively reproduce the C(1s) → π* band splitting in condensed polyacenes.^22^ Recently, core-level states of indole and some of its derivatives were studied by applying DFT method for inner-shell (IS) states.^12,13^ Besides indole, adenine and guanine were also studied.^10,11^

Over the past years, post Hartree–Fock methods applied to IS states have become very popular, leading to several developments. Different strategies at the coupled cluster level of theory were proposed, namely, linear response (LR)^23^ and equation-of-motion (EOM).^24^ An efficient approximation for core-excited states is the core-valence separation (CVS) proposed by Cederbaum,^25^ resulting in the CVS-CC methods.^24,26,27^ A lower-cost approach within CVS approximation is the combination with ADC, i.e., the CVS-ADC(2) method.^28,29^ Despite their great popularity, one drawback of the single reference methods is the inability to describe electronic transitions involving states with a multiconfigurational character.

Multireference (MR) methods can treat excited states with single and multiconfigurational character simultaneously.^30^ One strategy to converge core-states is the execution of the MCSCF procedure with restricted occupations for IS states (IS-MCSCF). In this protocol, it is possible to avoid the variational collapse of the wave function to the ground or to a low-lying excited state.^31−35^ It is also worth mentioning that, recently, LR-CASSCF^36^ and MR-CVS-ADC(2)^37^ methods were proposed for MR and strongly correlated systems, thus, increasing the number of protocols based on MR methods to inner-shell states.

The study of the carbon K-edge of the heterocycles and, in some cases, the nitrogen K-edge also brings an opportunity to raise a question of more fundamental importance, which is the localization of the core-hole in equivalent or almost equivalent atoms. In order to put things in perspective, it is worth discussing the very nature of the IS states. By applying NEXAFS spectroscopy, it is possible to infer the chemical environment. For instance, for carbon in a reduced environment, i.e., bonded to hydrogen or to other carbon atom, as in hydrocarbons, the first transition will lie at about 284 eV,^38^ while in an oxidized environment, such as CO_2_, the first transition lies at 290 eV.^32^ This can be qualitatively rationalized in terms of molecular orbital energies by invoking Koopmans’ theorem. In CO, for instance, the first molecular orbital corresponds to the oxygen K-edge, while the second corresponds to the carbon K-edge.^31^ In cases where the atoms are equivalent, such as N_2_,^33^ the states, although localized, combine to generate two almost degenerate, Σ_*u*_ and Σ_*g*_, states. An interesting case is N_2_O,^31^ where the nitrogen atoms are not equivalent. The first molecular orbital corresponds to the oxygen edge, the second orbital corresponds to the central, more oxidized, nitrogen, and the third corresponds to the terminal, less oxidized, nitrogen. How far can this analysis go? What would happen in cyclic molecules like the ones studied here?

**Figure 1:**
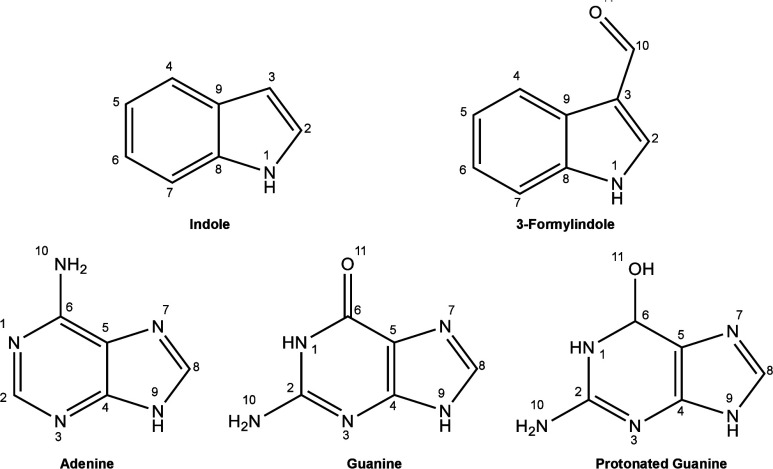
All molecules studied in this work include indole, adenine, 3-formylindole (3-FI), guanine, and protonated guanine.

In this work we have applied the inner-shell MCSCF method and compared it with the straightforward single reference method, TDDFT, for the simulation of the first preionization bands of the NEXAFS spectra at C, O, and N K-edges of the following biologically relevant nitrogen-bearing heterocycles; indole, 3-formylindole (3-FI), adenine, and guanine, all show in Figure 1. Also, for the TDDFT computations, we applied the direct vibronic coupling (DVC) method to analyze the vibronic coupling contribution.^38,39^ Furthermore, a comparison with experimental and theoretical results is presented when available.

## Computational Details

Calculations done at the MCSCF level for inner-shell states (IS-MCSCF) were performed using Molpro software. The basis set used was aug-cc-pVTZ for all but hydrogen atoms, for which aug-cc-pVDZ was used. This level of calculation for IS states takes into account an important feature of those states, which is the relaxation of valence orbitals due to the core hole. That is, the valence orbitals are optimized with a missing electron in the core orbital.

In the present case, all π electrons of the heterocycle valence have been included, including an electron of the core at the time. Hence, one calculation was independently done for each core orbital, with the same valence space. For instance, an indole has ten π electrons, and the valence active space is a CASSCF(10,10). The molecule contains 8 carbon atoms and 1 nitrogen, so there are 9 core orbitals, the first for nitrogen and the others for carbon atoms. Hence, we have performed 9 calculations, in which one of these, the respective inner-shell orbital, is included in the active space. A state-averaged calculation was done given the same weight to the ground state and the first IS state, but the transition moments and energies were calculated for more transitions from the ground to the IS states. The transitions for the nine core states were added in order to simulate the spectrum.

Similar procedures were performed for the other heterocycles. Accordingly, adenine has 10 core orbitals and 12 π electrons, 3-FI has 11 core orbitals and 12 π electrons, and finally, guanine has 11 core orbitals and 14 π electrons.

All TDDFT calculations were performed with Gaussian 2016 software^40^ using the PBE0 functional and aug-cc-pVTZ basis set and the relativistic approximation Douglas–Kroll–Hess for the core electrons.^41,42^ To help the comparison among methods, all spectra were normalized to 1. For all cases, vibronic coupling was considered using the direct vibronic coupling (DVC) method, which can be found in detail elsewhere.^39^ The broadening of all spectra was done with Gaussian functions with a full width at half-maximum (fwhm) of 0.2 eV.

## Results and Discussion

### Indole

**Figure 2:**
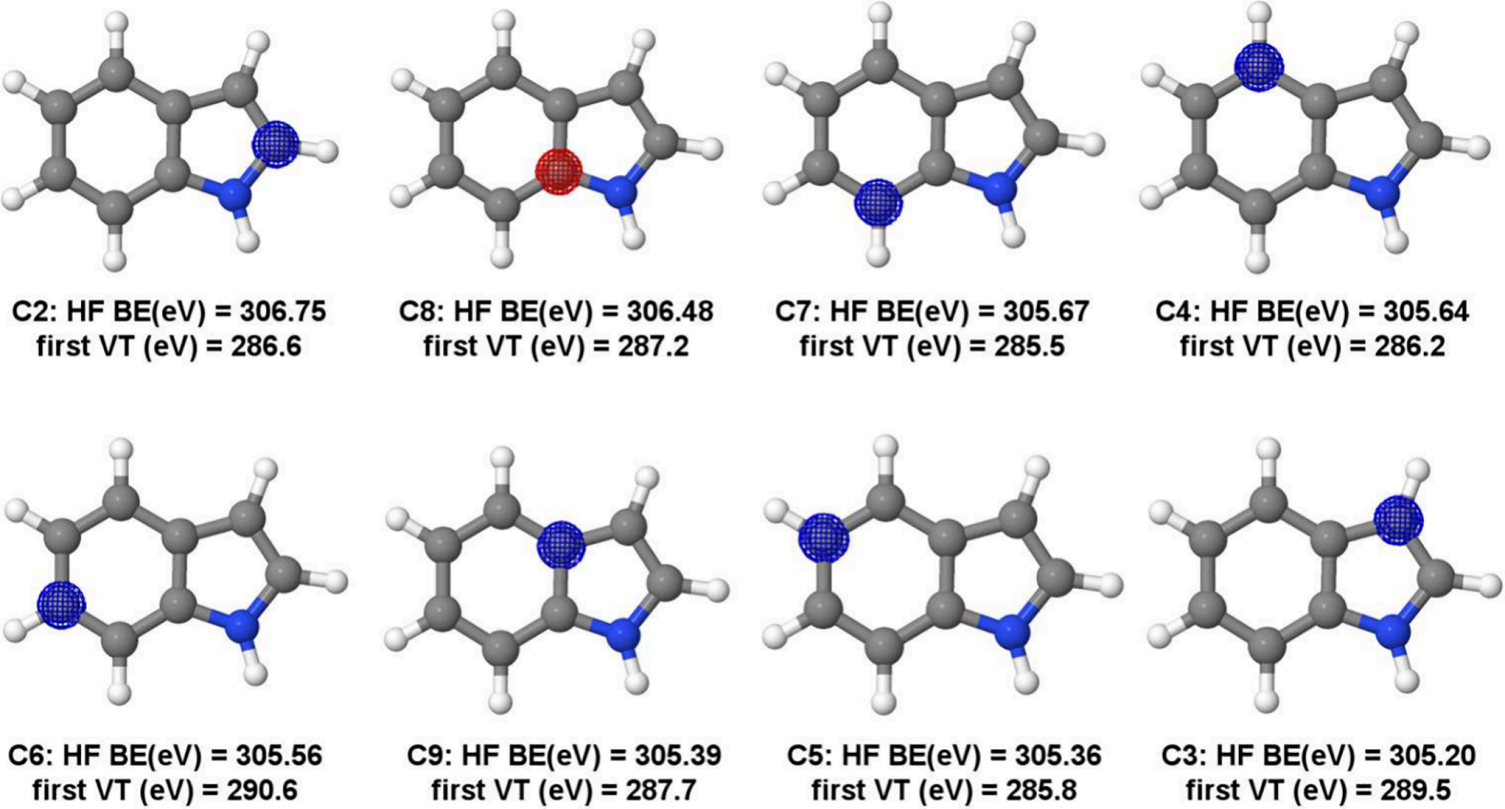
Hartree–Fock (HF) orbitals, HF binding energies (BE), and the first MCSCF vertical transitions (VE) of indole carbon K-edge.

**Figure 3:**
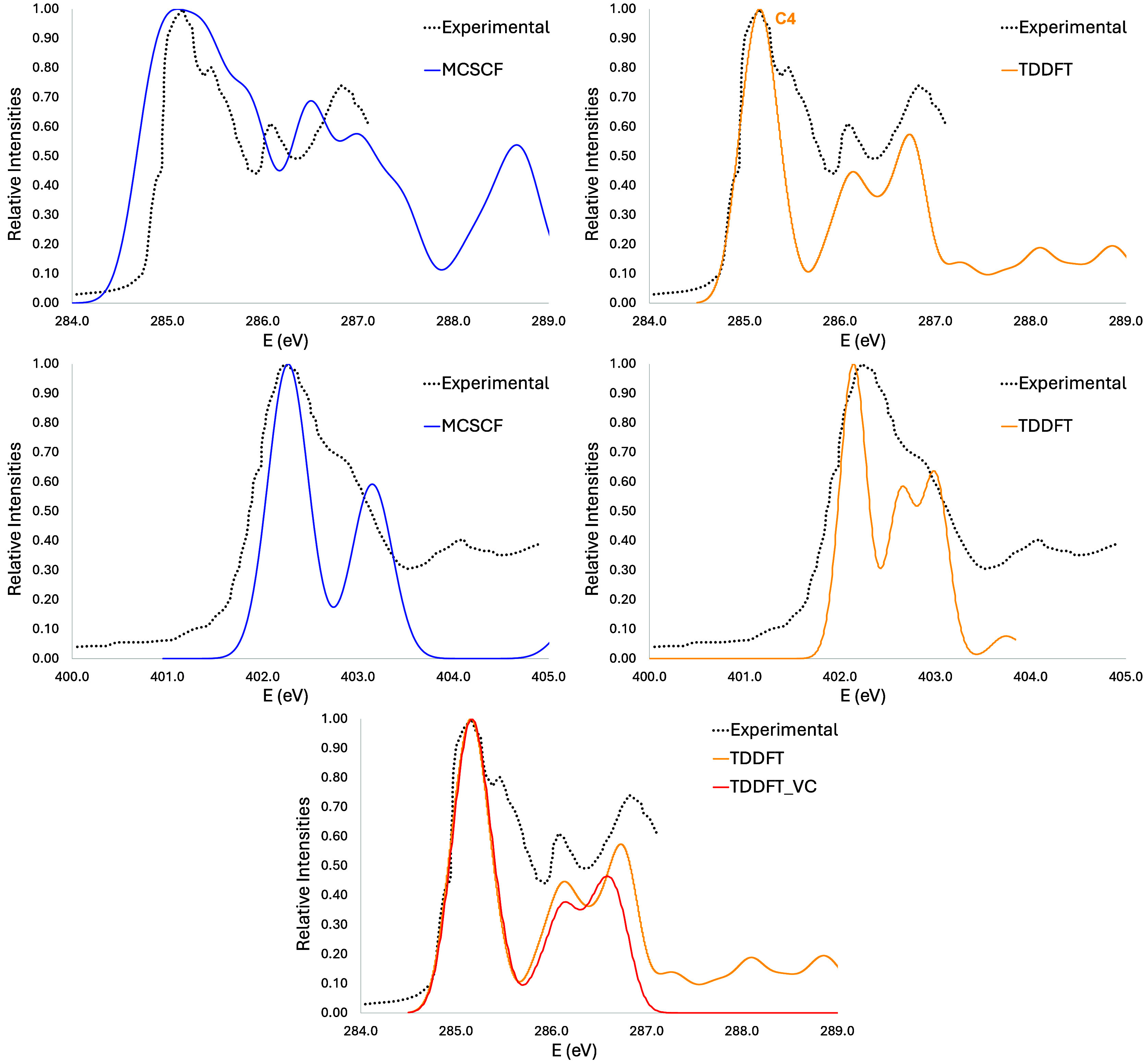
Indole K-edge vertical simulated spectra at carbon (top) and nitrogen (middle). A comparison of the vertical simulated spectra with the one obtained by considering vibronic coupling effects at the carbon edge is also shown (bottom). Simulated spectra have been obtained at two levels of theory, i.e, MCSCF and TDDFT.

All carbon inner-shell molecular orbitals of indole are present in Figure 2, and the vertical simulated spectra at the carbon (top) and nitrogen (middle) K-edge are presented in Figure 3. A comparison of the vertical simulated spectra with the one obtained by considering vibronic coupling effects at the carbon edge is also shown (bottom) in Figure 3. The red and blue curves are the simulated spectra built by a Gaussian broadening. The experimental data obtained by Ponzi and co-workers are also shown (black dots).^13^

The agreement is especially good for MCSCF for the first bands of the preionization edge. These transitions concern excitation coming from all orbitals forming the carbon edge, calculated one at a time, as explained above. In this case, the orbitals involved are 2–9. The spectra at the nitrogen K-edge are also shown in Figure 2. The agreement with the experiment is quite satisfactory for the first experimental band. Above 404 eV, the Rydberg character of the states turns the simulations more challenging.^21^ It is worth emphasizing that all IS-MCSCF simulated spectra are done from a vertical calculation with no explicit consideration of vibronic coupling, which is discussed later in this section. The spectra were displaced at 0.70 and 0.25 eV for carbon and nitrogen edges, respectively.

When it comes to the TDDFT technique, there is still good agreement with the experimental spectra for both edges. For the carbon edge, three major bands can be seen in the spectra. The first one is composed of six transitions, all of them from carbon 1s orbitals to the nitrogen, with the major contribution from C4. These are, in fact, the main contributions for the third and fourth band. The second experimental band is missing from the simulated spectrum, but it can be seen at the MCSCF level. In fact, MCSCF gives better agreement for the first band, but the intensities of bands 3 and 4 are reversed. TDDFT, however, can predict these intensities correctly. As for the nitrogen K-edge, the agreement is also quite good with TDDFT, although the intensities are better described by MCSCF. It is worth mentioning that these TDDFT spectra reported were shifted by a 9.5 eV factor for the carbon edge and 11.35 eV for the nitrogen edge.

In Figure 2, all 1s core orbitals are present for nitrogen and carbon atoms in ascending order of the binding energy (obtained at the Hartree–Fock level) according to the numbering present in Figure 1. A simpler analysis based on the Koopmans’ theorem states that the energies for the first transition (or ionization) from each orbital will decrease from 2 to 9, also decreasing the binding energy (BE). The analysis done here shows a different picture, though. In Figure 2 the BE at the Hartree–Fock (HF) level, the orbitals, and the first vertical transition (VT) energy at the IS-MCSCF level are shown. As can be seen, contrary to what is expected, the larger BE does not necessarily correspond to the largest value of the first VT energy. The result can be rationalized by considering that relaxation of the valence orbitals is different depending on the orbital involved in the core-hole.

The electron in the 1s orbital of carbon atom number 6 (C6) exhibits the larger first VT energy (290.6 eV), despite not presenting the highest BE (305.56 eV). Moreover, BE values are in a different order than transition values, emphasizing the importance of the orbital relaxation. For instance, C2 and C8 have the highest BE values of 306.75 and 306.48 eV, respectively, but the first VT energies are not the highest ones (286.6 and 287.2 eV). From the spectrum analysis, 1s orbitals localized at C2 and C8 carbon atoms do not contribute for the first band (from 284 to 286 eV), but contribute significantly to the one in between 286 and 288 eV, in agreement with previous TDDFT results.^13^

In order to consider vibronic coupling by the DVC method, a series of calculations are done along the normal coordinates, which means the method is computationally intense. That is why they were done only at the TDDFT level. Unfortunately, all this effort has not been rewarded, since the spectrum corrected with vibronic coupling has not shown any significant difference when referred to the vertical one. This is shown in the bottom panel of Figure 3 for the carbon edge. The nitrogen edge is shown in the SI.

### 3-Formylindole

**Figure 4:**
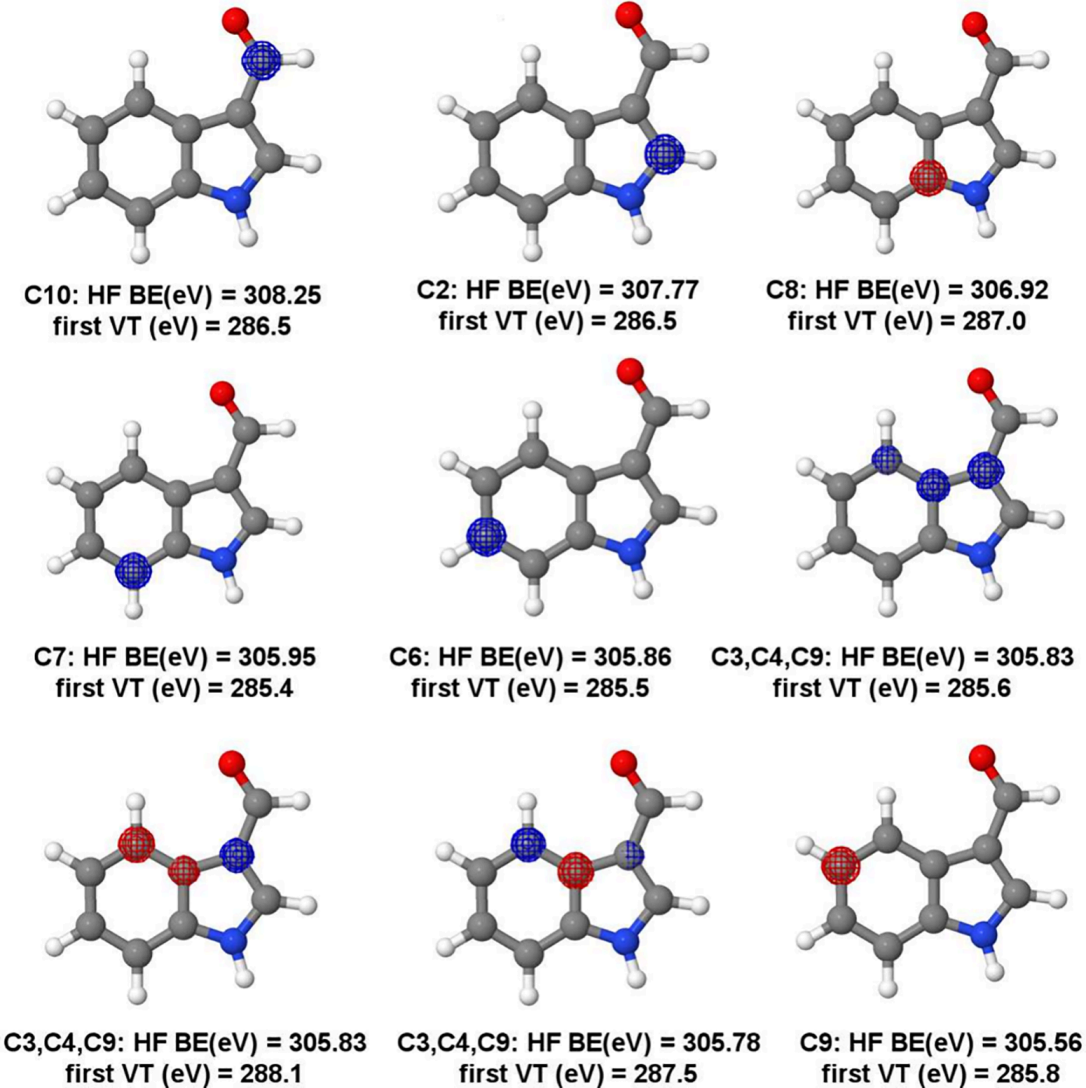
Hartree–Fock (HF) orbitals, HF binding energies (BE), and first MCSCF vertical transitions (VE) of 3-formylindole (3-FI) carbon K-edge.

In the case of 3-FI, the highest values of BEs in the carbon K-edge were obtained for C10 (CHO group) and C2 (308.25 and 307.77 eV), but the larger first VT energies reported here were obtained for the delocalized 1s orbitals involving C3, C4, and C9 carbon atoms (Figure 4).

**Figure 5:**
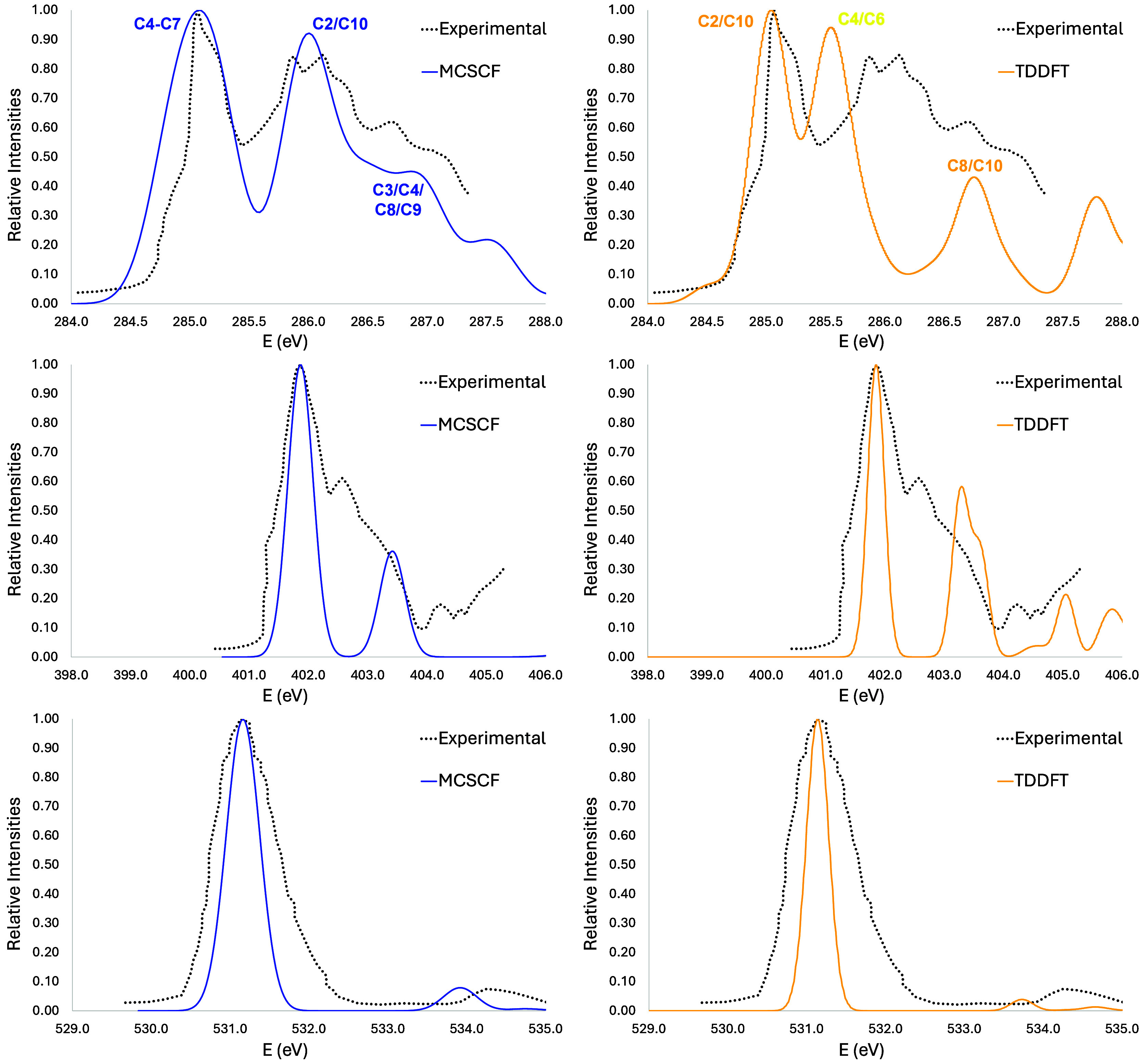
3-Formylindole (3-FI) vertical simulated spectra at carbon (top), nitrogen (middle), and oxygen (botton) K-edges. Simulated spectra have been obtained at two levels of theory, i.e., MCSCF (blue line) and TDDFT (red line).

The simulated NEXAFS at the same edge for MCSCF is shown in Figure 5. The agreement with experimental results is good. The first band (from 284 to 285.5 eV) is mainly composed by transitions from C4–C7 1s orbitals, in agreement with previous theoretical results.^13^ The main contributions to the second band around 286 eV are transitions from 1s orbitals localized on C10 (CHO group) and C2. The most important transitions for the formation of the band shoulder above 286 eV and below 288 eV are from C3, C4, C8, and C9 carbon atoms. Above 288 eV there are several states with Rydberg character.^13^ From the analysis of the nitrogen K-edge spectrum, the main band is composed of two transitions (Figure 5, middle panel). A similar theoretical result was obtained by Ponzi and co-workers.^13^ Turn our attention to the oxygen K-edge spectrum, where one can see the tree transitions in Figure 5. From the experimental result analysis, it is possible that water molecules interfered with the result.^13^ Nevertheless, it is possible to see three independent bands from the 3-FI simulated NEXAFS spectrum.

For the carbon edge, TDDFT gives the worst agreement of all of the cases studied here. The relative energies are poorly predicted, and the most intense band is missing. The first two bands, which are very close in energy, are composed of nine transitions. The major contributions for these are from C2 and C10 and from C4 and C6. The smallest band in the simulated spectrum also has major contributions from C10 and C8. As one can see, the major contributions come from the carbons adjacent to a heteroatom. It is worth mentioning that the delocalized orbitals obtained with MCSCF are not present in the DFT result. All DFT orbitals are localized in only one atom. For the nitrogen edge, however, two experimental bands are present, but the relative energies are still slightly different, in agreement with MCSCF. Finally, for the oxygen edge, the agreement is better, as there is only one band on the experimental spectrum, which was obtained with the TDDFT method. The displacements made were 9.459, 11.54, and 12.8 eV for carbon, nitrogen, and oxygen, respectively, for TDDFT and 0.52, 1.12, and 2.5 eV for MCSCF.

### Adenine

**Figure 6:**
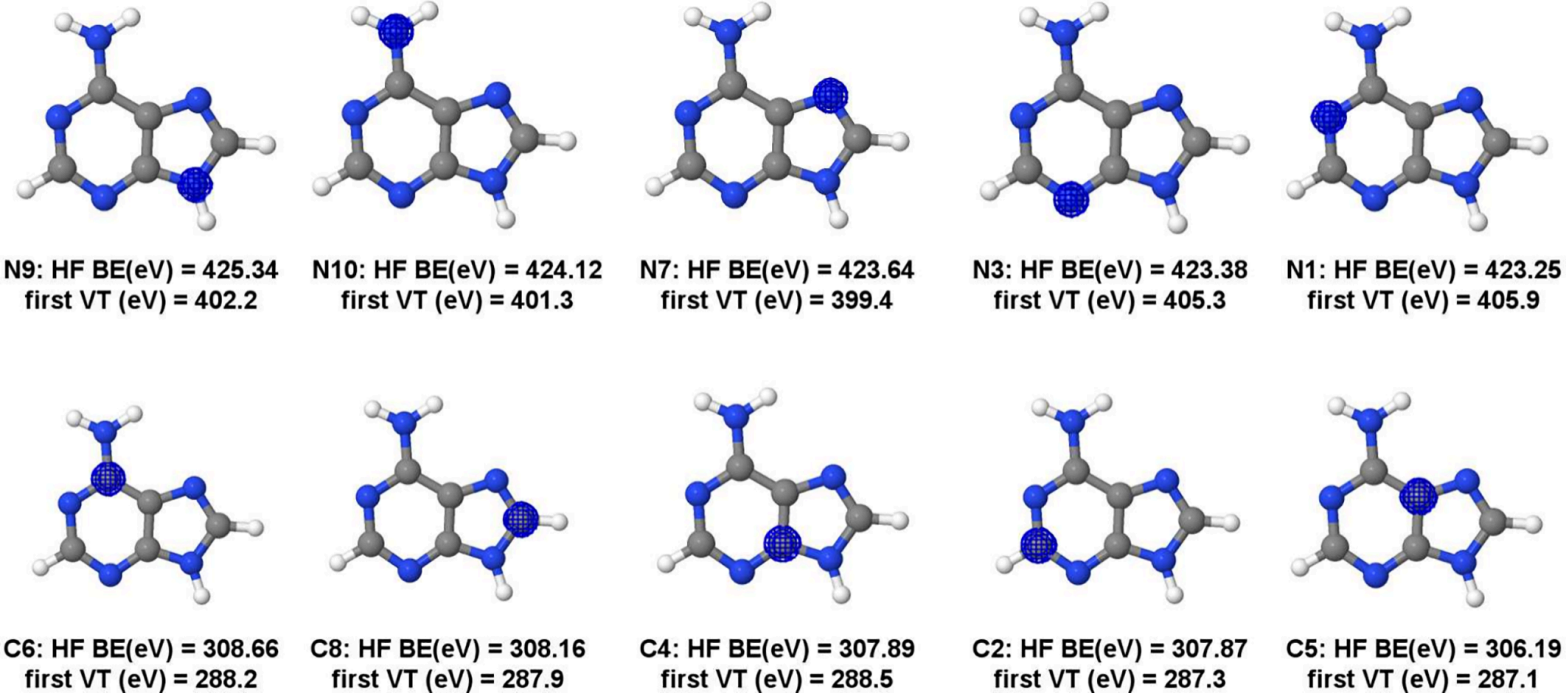
Hartree–Fock (HF) orbitals, HF binding energies (BE), and first MCSCF vertical transitions (VE) of adenine carbon and nitrogen K-edges.

For adenine, the electrons with the highest BEs are localized in the N9 and N10 nitrogen atoms (425.34 and 424.12 eV), but the first VT energies are not the highest ones (402.2 and 401.3 eV). Moreover, the largest first VT energies were obtained for electrons localized in N3 and N1 1s orbitals (405.3 and 405.9 eV), see Figure 6. Similar trends occur for the carbon K-edge. While the highest BE was obtained for the electron in the 1s orbital of carbon C6, the larger first VT energy was obtained for the electron in the 1s orbital of C4. In general, BE and the first VT energies are in different orders (see Figure 6).

**Figure 7:**
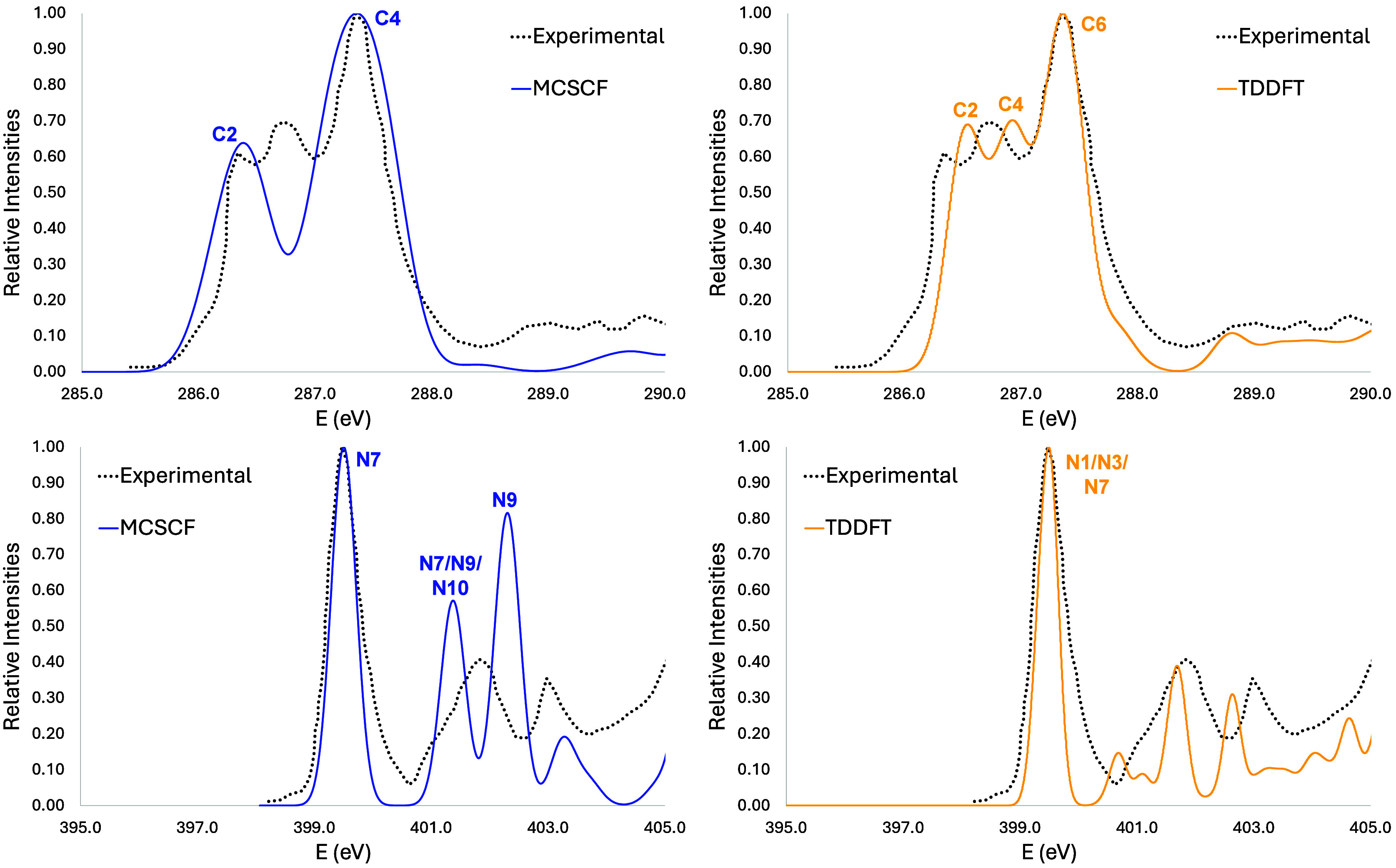
Adenine vertical simulated spectra at carbon (top) and nitrogen (botton) K-edges at two levels of theory: MCSCF (blue line) and TDDFT (red line).

Concerning the NEXAFS spectrum on the carbon K-edge (Figure 7, top panel), the first experimental band has three peaks between 285 and 288 eV.^10^ Although the second transition appears a little below the second peak, our simulated spectrum is in reasonable agreement. The first two transitions are due to C2 and C5 carbon atoms, and the only orbital that does not contribute to the first band is the 1s localized on C4 and C6. For the nitrogen K-edge (Figure 7, bottom panel), the first band is due to the transition from the 1s orbital localized at N7 nitrogen atom. The second one is composed by three transitions from N10 (in the NH_2_ group, 401.3 eV), N7 (402.1 eV), and N9 (402.2 eV), respectively, but in our simulation it appears as two bands instead an asymmetric one, such as in the experimental spectrum.^10^ The third band is mostly composed by the transition from N9 (403.2 eV). It is worth noting that the results obtained with CVS-ADC(2) and 6-31+G basis sets generate a more complex simulated NEXAFS spectrum,^10^ making the comparison difficult with our results.

For both carbon and nitrogen edges, the agreement between TDDFT and the experimental results is very good, even better than MCSCF. The major contributions to the three main bands are, respectively, C2, C4, and C6. One may notice again that all atoms that contribute are the carbons adjacent to heteroatom and those carbon atoms that are in between two nitrogen atoms. As for the nitrogen edge, the first band is composed by three transitions with almost the same intensity, with contributions from N1, N3, and N7. The displacements made were 9.85 and 10.83 for carbon and nitrogen, respectively, for TDDFT and 0.88 and 0.08 eV for MCSCF.

### Guanine

**Figure 8:**
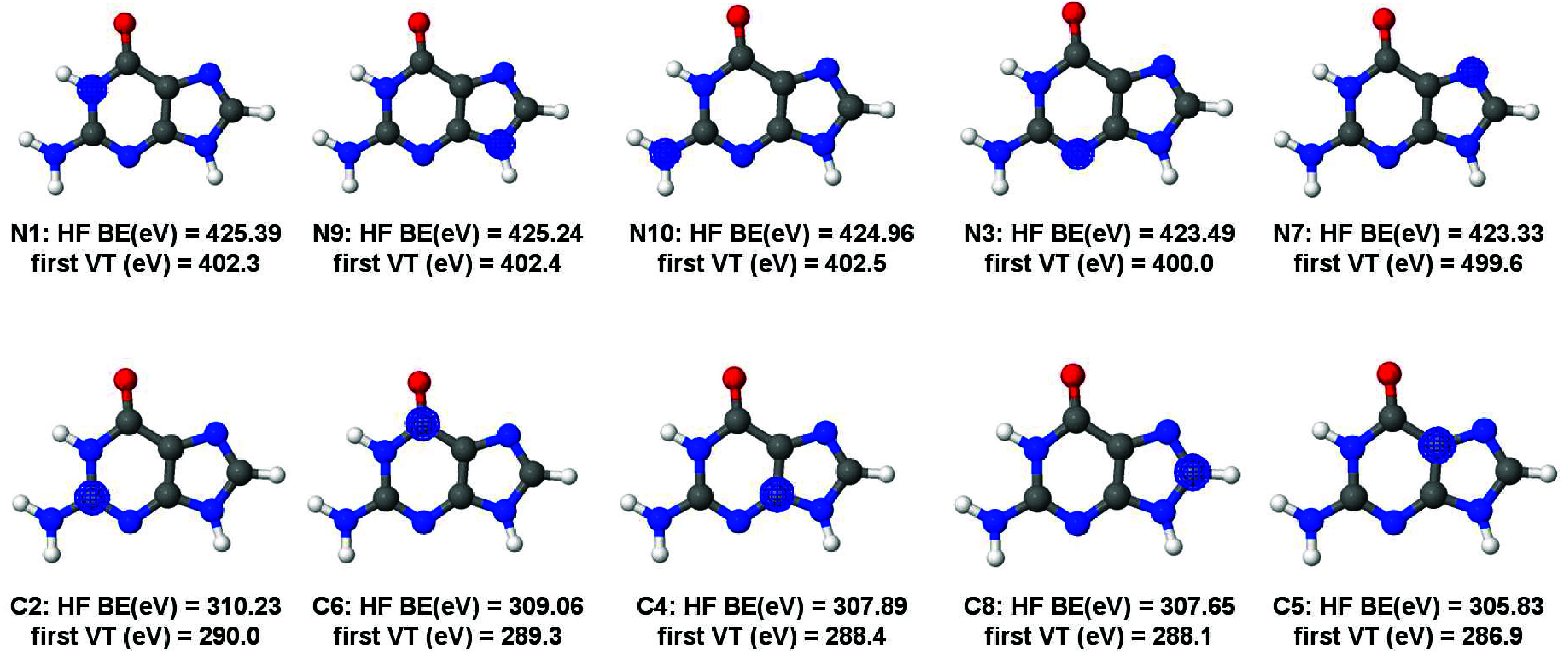
Hartree–Fock (HF) orbitals, HF binding energies (BE), and first MCSCF vertical transitions (VE) of the guanine carbon and the nitrogen K-edges.

**Figure 9:**
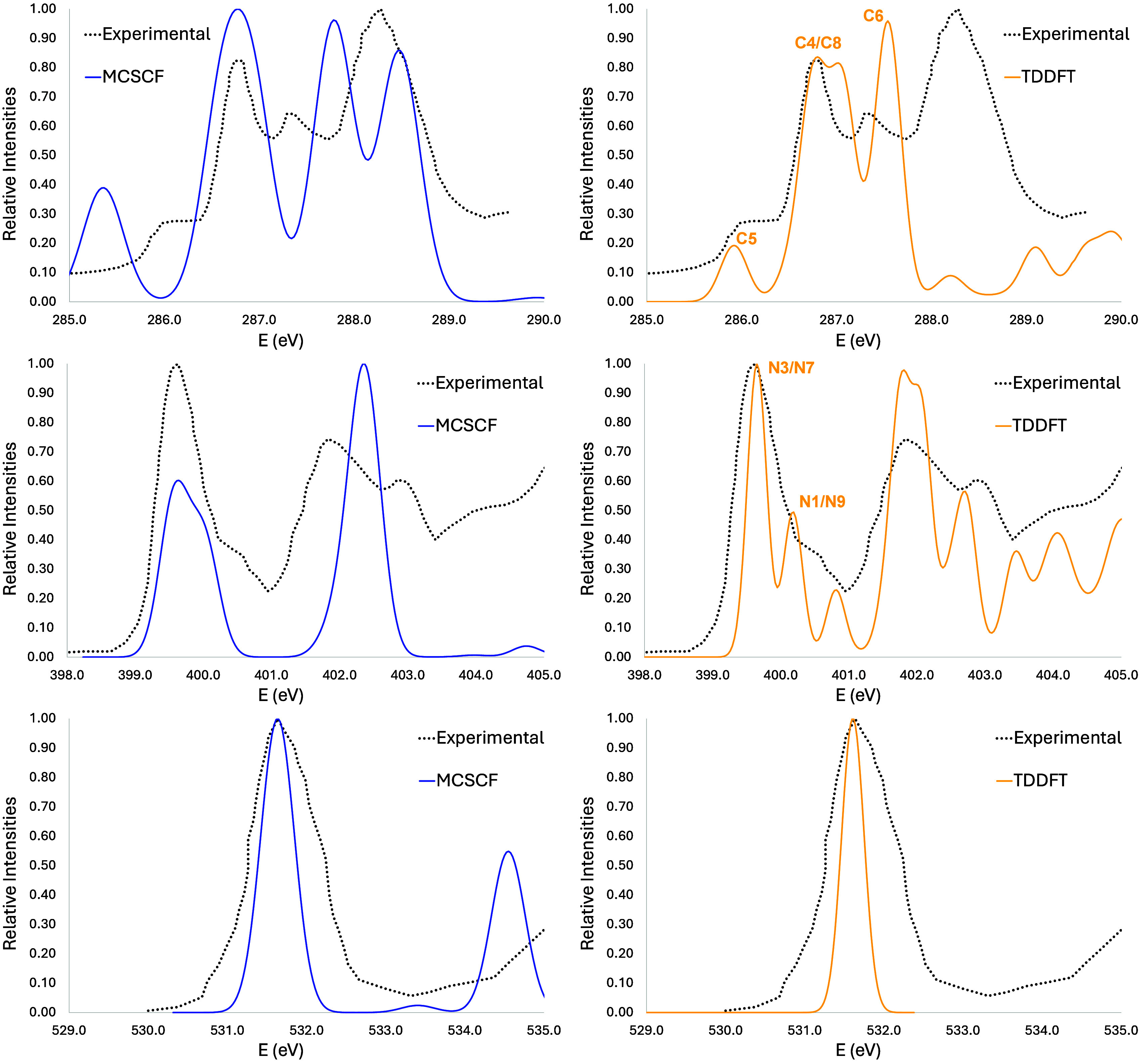
Guanine vertical simulated spectra at carbon (top), nitrogen (middle), and oxygen (bottom) K-edges at two levels of theory, MCSCF (blue line) and TDDFT (red line). For the oxygen K-edge spectrum, two tautomers were considered. The first band is due to the 1s orbital from the carbonyl group, but the second (experimental) band is due to the 1s orbital from the protonated oxygen in the OH group.

In order to study the inner-shell states of guanine, we chose one representative low-energy tautomer as a case study (Figure 1). For the carbon and nitrogen K-edge, we obtained the same energetic order for BEs and first VT energies, as shown in Figure 8. The NEXAFS spectrum for the carbon K-edge (Figure 9, top panel) has a complex feature probably due to the different contributions from the tautomers.^11^ However, the first experimental band (below 290 eV) is reasonably simulated and had contributions from all carbon atoms. Above 290 eV, the excited states have a strong Rydberg character, making the theoretical result less accurate.^11^ The first experimental band in the nitrogen K-edge spectrum (Figure 9, middle panel) is composed of two transitions from 1s orbitals localized at N3 and N7, which are pyridinic nitrogen atoms (C—N=C motif). The second band is not as well described as the first one, and it is composed of several transitions, in agreement with previous theoretical simulations, and above 404 eV there are several states with Rydberg character.^11^ The first band in the oxygen K-edge spectrum is well reproduced with both methods, but the second (experimental) band is due to the 1s orbital from protonated oxygen in the OH group. At the MCSCF level, we computed the K-edge oxygen spectra for two tautomers, one of them with the OH group. The final spectrum is the sum of both individual ones. From Figure 9, bottom left panel, it is clear that the second band was obtained in the final simulated spectrum.

**Figure 10:**
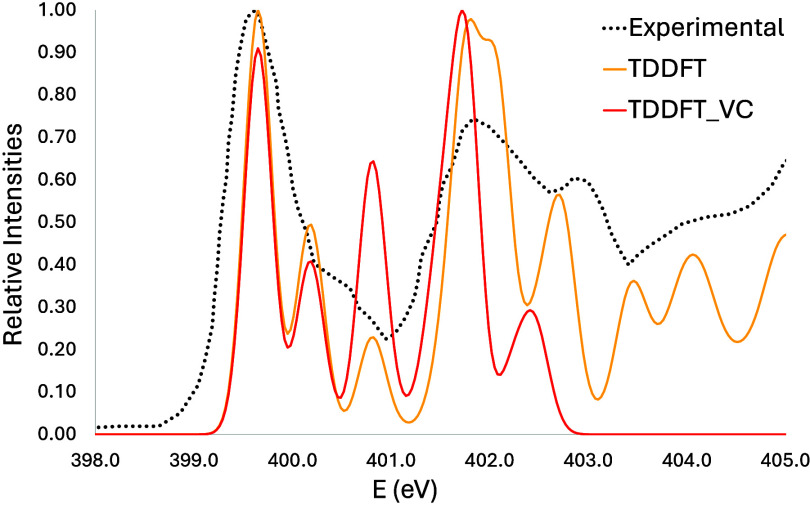
Guanine nitrogen K-edge simulated spectra at the TDDFT level of theory with vibronic coupling.

From the TDDFT results, the carbon edge has one missing band, but the first three bands seem reasonable. The first and smallest one has contributions from C5, while the following two have contributions from C4, C8, and C6, respectively. The nitrogen edge shows two bands but with relative intensities, different than experimental ones. Yet, between these two, there is a third band apparently not seen on the experimental spectra, and the intensity increases even more with vibronic coupling, as seen in Figure 10. It can be due to a bad prediction on the energy of the transition or to some physical effect affecting the experimental spectra, such as the tautomer mentioned before. The first band has contributions from N3 and N7, in accordance with the results seen with MCSCF, and the second band has contributions from N1 and N9. Lastly, like the MCSCF result, the first band of the oxygen edge shows good agreement with the experimental one, but the second band is missing, as it is caused by a different tautomer (containing an OH group). The displacements made were 9.50, 11.00, and 12.40 for carbon, nitrogen, and oxygen, respectively, for TDDFT and 1.5, 0.00, and 0.95 eV for MCSCF.

## Conclusion

In this work, we simulated the NEXAFS spectra of representative nitrogen-bearing heterocycles by applying IS-MCSCF and TDDFT (PBE0 functional) methods. The influence of vibronic coupling was also investigated at the TDDFT level of theory. Remarkably, the Hartree–Fock binding energies, which are commonly used by experimentalists to easily obtain a qualitative picture of the spectrum, are not in agreement with the vertical energy order obtained by the IS-MCSCF method. The difference in relative vertical energies between the two approaches can be as large as 3 eV. Concerning the comparison between IS-MCSCF and TDDFT, in most cases the results are very similar and in good agreement with experimental results. The most complicated case was guanine, due to the variety of possible tautomers. However, we were able to conclude, within the IS-MCSCF method, that the oxygen K-edge split due to protonation by forming the OH group. Vibronic coupling and vertical transition results were very similar for all but the guanine nitrogen K-edge. In this case, a new band appears, induced by vibronic coupling. Finally, to the best of our knowledge, it is the first time that the IS-MCSCF method has been successfully applied to large molecules such as bicyclic heterocycles.

## Supporting Information Available

Molecular geometries, protonated guanine, and vibronic coupling results (PDF)
