# Supplementary material for: NEXAFS Spectra Simulations of Nitrogen-Bearing Heterocycles
Source: ACS Omega. 2024 Oct 16;9(43):43884–93. doi: 10.1021/acsomega.4c07024 (PMC11525529; doi:10.1021/acsomega.4c07024)
Supplement: Supplementary file 1 — ao4c07024_si_001.pdf [file ao4c07024_si_001.pdf]

Supporting Information: NEXAFS Spectra Simulations of Nitrogen-bearing Heterocycles

Ricardo R. Oliveira,* Amanda D. Torres, and Alexandre B. Rocha*

Chemistry Institute, Federal University of Rio de Janeiro, Rio de Janeiro, Brazil - 21941-909

E-mail: rrodrigues.iq@gmail.com; rocha@iq.ufrj.br

Contents

1 Optimized Geometries **S-3**

2 Vibronic Coupling results **S-7**

3 Guanine results **S-8**

1 Optimized Geometries

Table S1: Optimized geometry for adenine. The calculations were performed with DFT/PBEh1PBE method and aug-cc-pVTZ using Gaussian16.

N	-1.8671360000	-1.7838250000	0.0000010000
N	2.0604960000	0.5815650000	0.0000000000
N	1.1735090000	-1.4664200000	-0.0000020000
N	0.0235420000	1.9095360000	0.0000000000
N	-1.9326770000	0.5240080000	0.0000000000
C	2.2736400000	-0.7707750000	0.0000010000
C	-1.2222300000	-0.6050540000	0.0000010000
C	0.1778530000	-0.5171990000	0.0000000000
C	0.7053070000	0.7680350000	0.0000000000
C	-1.2838000000	1.6893160000	-0.0000020000
H	-1.3496810000	-2.6417710000	-0.0000020000
H	-2.8694110000	-1.7924060000	-0.0000010000
H	3.2705540000	-1.1845820000	0.0000020000
H	2.7550750000	1.3070030000	-0.0000020000
H	-1.9152920000	2.5717700000	0.0000050000

Table S2: Optimized geometry for guanine. The calculations were performed with DFT/PBEh1PBE method and aug-cc-pVTZ using Gaussian16.

O	-0.2029880000	2.6697170000	-0.0015610000
N	-2.9509780000	-1.0053960000	-0.0615930000
N	1.7241280000	-1.4906680000	-0.0026990000
N	2.2105400000	0.6805690000	0.0066450000
N	-0.6870470000	-1.4315860000	0.0057140000
N	-1.4667750000	0.7800380000	-0.0042110000
C	2.6983720000	-0.5223940000	0.0012930000
C	-0.2162380000	1.4605590000	0.0020870000
C	0.8497400000	0.5037590000	0.0057010000
C	0.5277560000	-0.8445380000	-0.0007340000
C	-1.6597890000	-0.5657460000	-0.0035970000
H	-2.2591370000	1.4011110000	-0.0602970000
H	-3.0531400000	-1.9908600000	0.1099030000
H	-3.6704480000	-0.4271180000	0.3348700000
H	3.7474740000	-0.7743770000	0.0003510000
H	1.8510460000	-2.4870320000	-0.0078300000

Table S3: Optimized geometry for indole. The calculations were performed with DFT/PBEh1PBE method and aug-cc-pVTZ using Gaussian16.

N	-1.0858220000	1.5471630000	0.0000000000
C	-2.2350350000	0.7987050000	0.0000000000
C	-1.9184130000	-0.5261200000	0.0000000000
C	0.4221740000	-1.6717130000	0.0000000000
C	-0.4920080000	-0.6138680000	0.0000000000
C	0.0000000000	0.7113270000	0.0000000000
C	1.3607630000	0.9993200000	0.0000000000
C	2.2372210000	-0.0670870000	0.0000000000
C	1.7722150000	-1.3901000000	0.0000000000
H	-1.0480110000	2.5483550000	0.0000000000
H	-3.2004740000	1.2783130000	0.0000000000
H	-2.6220160000	-1.3423230000	0.0000000000
H	1.7229050000	2.0203720000	0.0000000000
H	3.3035120000	0.1209520000	0.0000000000
H	0.0737510000	-2.6974710000	0.0000000000
H	2.4895970000	-2.2011260000	0.0000000000

Table S4: Optimized geometry for 3-formylindole. The calculations were performed with DFT/PBEh1PBE method and aug-cc-pVTZ using Gaussian16.

O	3.5841590000	-0.5720080000	0.0000000000
N	-0.4656330000	-1.8289900000	0.0000000000
C	2.5898190000	0.1206620000	0.0000000000
C	0.8827110000	-1.7112930000	0.0000000000
C	1.2304040000	-0.3808650000	0.0000000000
C	-0.3243280000	1.7249080000	0.0000000000
C	0.0000000000	0.3661870000	0.0000000000
C	-1.0460490000	-0.5787660000	0.0000000000
C	-2.3865610000	-0.2164190000	0.0000000000
C	-2.6758970000	1.1337360000	0.0000000000
C	-1.6547860000	2.0923880000	0.0000000000
H	2.6827650000	1.2252440000	0.0000000000
H	-0.9667960000	-2.6982790000	0.0000000000
H	1.5300720000	-2.5730960000	0.0000000000
H	-3.1750310000	-0.9589490000	0.0000000000
H	-3.7089280000	1.4570140000	0.0000000000
H	0.4502110000	2.4813170000	0.0000000000
H	-1.9180140000	3.1425030000	0.0000000000

2 Vibronic Coupling results

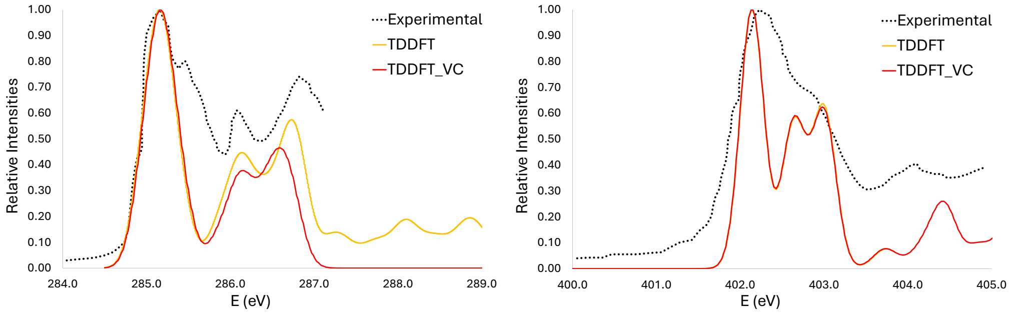

Figure S1: Indole K-edge simulated spectra at TDDFT level of theory with vibronic coupling.

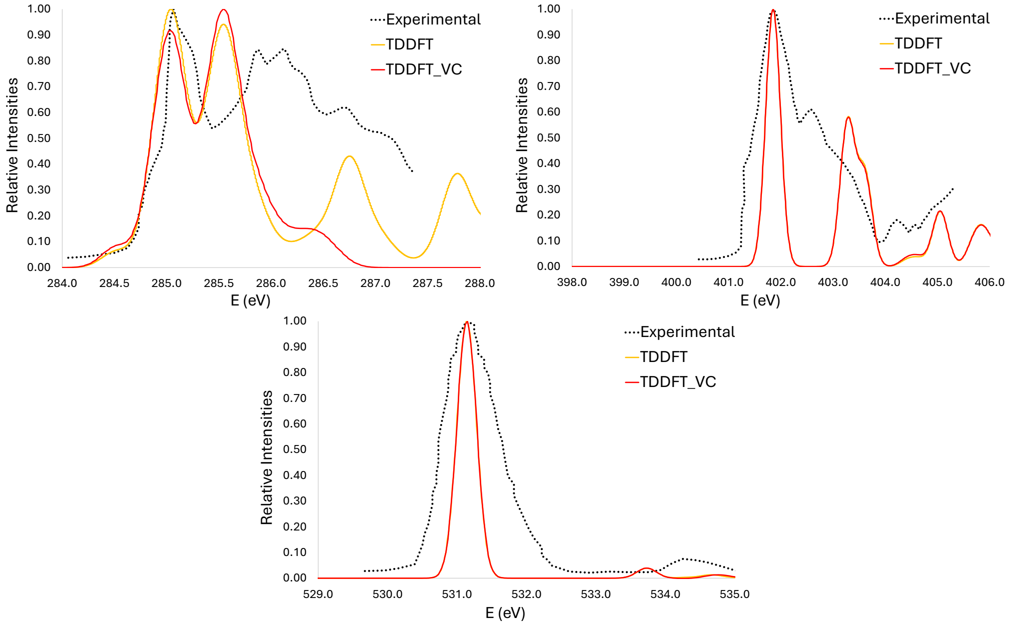

Figure S2: 3-formylindole (3-FI) K-edge simulated spectra at TDDFT level of theory with vibronic coupling.

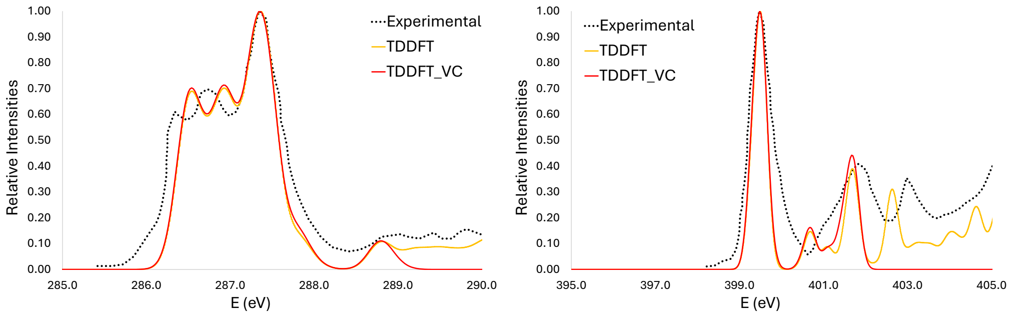

Figure S3: Adenine K-edge simulated spectra at TDDFT level of theory with vibronic coupling.

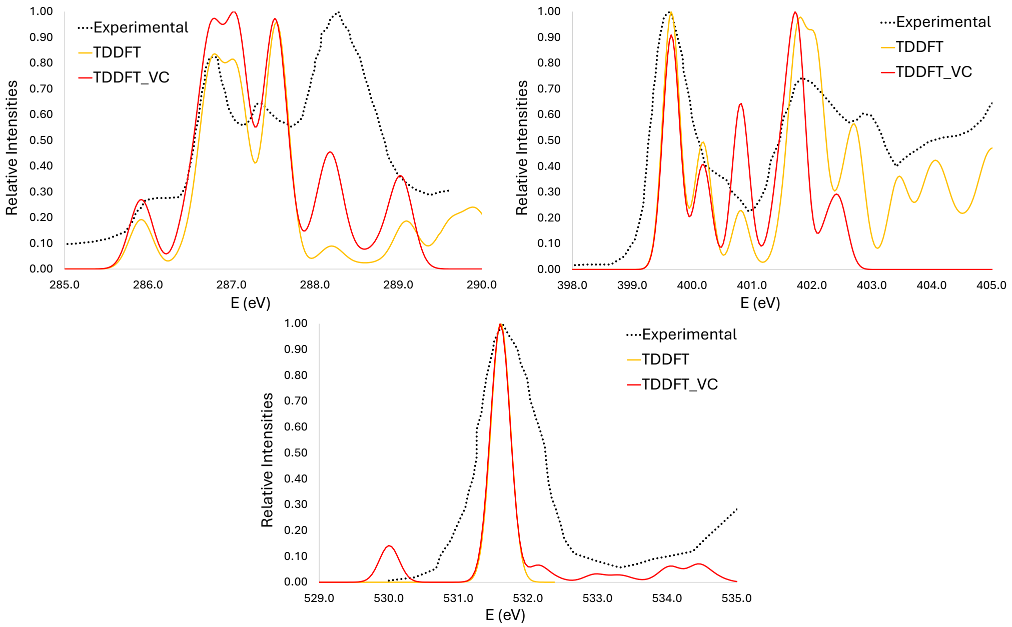

Figure S4: Guanine K-edge simulated spectra at TDDFT level of theory with vibronic coupling.

3 Guanine results

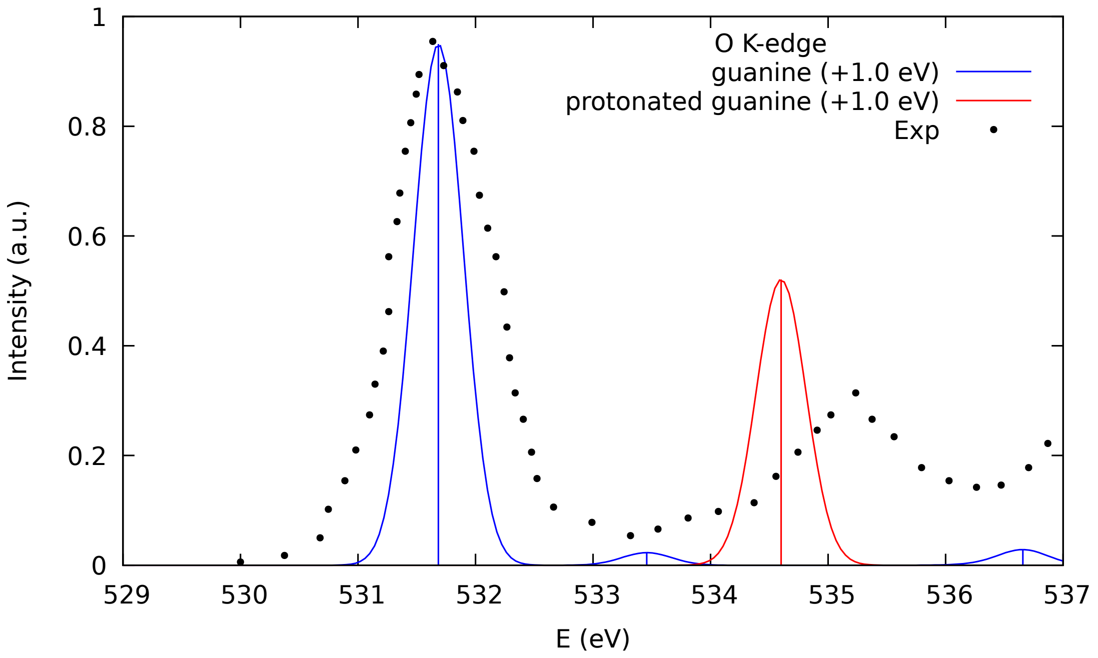

Figure S5: Guanine and protonated guanine Oxygen K-edge simulated spectra at MCSCF level of theory.
